# Abnormal Brain Connectivity Patterns in Adults with ADHD: A Coherence Study

**DOI:** 10.1371/journal.pone.0045671

**Published:** 2012-09-26

**Authors:** João Ricardo Sato, Marcelo Queiroz Hoexter, Xavier Francisco Castellanos, Luis A. Rohde

**Affiliations:** 1 Center of Mathematics, Computation and Cognition, Universidade Federal do ABC, Santo Andre, Brazil; 2 Laboratório Interdisciplinar de Neurociências Clínicas; Department of Psychiatry, Universidade Federal de São Paulo, São Paulo, Brazil; 3 ADHD Outpatient Program, Child and Adolescent Psychiatric Division, Hospital de Clínicas de Porto Alegre, Porto Alegre, Brazil; 4 Phyllis Green and Randolph Côwen Institute for Pediatric Neuroscience, New York University Child Study Center, New York, New York, United States of America; 5 Instituto Nacional de Psiquiatria do Desenvolvimento, Porto Alegre, Brazil; Institution of Automation, CAS, China

## Abstract

Studies based on functional magnetic resonance imaging (fMRI) during the resting state have shown decreased functional connectivity between the dorsal anterior cingulate cortex (dACC) and regions of the Default Mode Network (DMN) in adult patients with Attention-Deficit/Hyperactivity Disorder (ADHD) relative to subjects with typical development (TD). Most studies used Pearson correlation coefficients among the BOLD signals from different brain regions to quantify functional connectivity. Since the Pearson correlation analysis only provides a limited description of functional connectivity, we investigated functional connectivity between the dACC and the posterior cingulate cortex (PCC) in three groups (adult patients with ADHD, n = 21; TD age-matched subjects, n = 21; young TD subjects, n = 21) using a more comprehensive analytical approach – unsupervised machine learning using a one-class support vector machine (OC-SVM) that quantifies an abnormality index for each individual. The median abnormality index for patients with ADHD was greater than for TD age-matched subjects (p = 0.014); the ADHD and young TD indices did not differ significantly (p = 0.480); the median abnormality index of young TD was greater than that of TD age-matched subjects (p = 0.016). Low frequencies below 0.05 Hz and around 0.20 Hz were the most relevant for discriminating between ADHD patients and TD age-matched controls and between the older and younger TD subjects. In addition, we validated our approach using the fMRI data of children publicly released by the ADHD-200 Competition, obtaining similar results. Our findings suggest that the abnormal coherence patterns observed in patients with ADHD in this study resemble the patterns observed in young typically developing subjects, which reinforces the hypothesis that ADHD is associated with brain maturation deficits.

## Introduction

Attention Deficit/Hyperactivity Disorder (ADHD) is a highly prevalent disorder in childhood and adolescence [1] contributing to substantial lifetime impairment [2]. It is a heterogeneous syndrome characterized by excessive inattention, hyperactivity and impulsivity [3] that tend to persist into adulthood. Until recently, most neurobiological studies in ADHD were performed during childhood/adolescence.

Based on the observations of executive dysfunctions in ADHD [4], [5], convergent data from functional neuroimaging studies have supported the involvement of fronto-striatal and mesolimbic circuitry in the pathophysiology of ADHD [6], [7]. Specifically, hypoactivation of the dorsolateral prefrontal cortex, inferior prefrontal cortex, dorsal anterior cingulate cortex, basal ganglia, thalamus and the particular regions of the parietal cortex were detected in ADHD compared to control subjects [8]. Although most fMRI studies focus on identifying brain regions which respond to certain stimuli, and in quantifying their relative activations, it is well established that even simple behaviours are products of interactions between nodes of complex and interconnected neural networks [9]. Thus, the comprehension and description of functional connectivity within the brain are crucial to enhance our understanding of cognitive processing or abnormal behaviour. Using simple correlation analysis, Biswal et al. [10] showed that intrinsic activity in different motor areas has similar time-courses, highlighting that such areas are functionally connected, even during resting state acquisitions. Based on consistent observations of deactivation during externally oriented cognitive tasks and activation during rest, Raichle et al. [11], [12] and Buckner & Vincent [13] described the brain’s Default Mode Network (DMN), which has become the most frequently studied intrinsic connectivity network, particularly in clinical populations.

Specifically, functional connectivity magnetic resonance imaging (fcMRI) has been used to investigate Alzheimer disease, depression, autism, epilepsy in comparison to subjects with typical development [14]. Resting state fcMRI can be acquired simply and can be obtained with relatively rapid protocols. In adults with ADHD, resting state fcMRI studies have suggested reduced connectivity in the DMN [15], [16]. In 2008, Castellanos et al. [16] proposed a new locus of dysfunction in ADHD, that had been implicated in attention lapses [17], by showing that patients with this disorder presented decreases in functional connectivity between the anterior cingulate and precuneus/posterior cingulate cortex (PCC) regions relative to subjects with typical development.

Most neuroimaging studies exploring functional connectivity are based on extracting Pearson or Spearman correlation coefficients between the signals from different brain regions. Correlation coefficients can be useful as an index of synchronous BOLD signal fluctuations measured in distinct areas. On the other hand, they may be strongly influenced by artifacts and do not take into account the temporal-scale of the dependence, i.e., short/long term or frequency features. Spectral coherence analysis (SCA) [18], [19] is a more informative approach in the sense that it is a dependence measure in the frequency domain. SCA makes it possible to measure the dependence between two time series at different frequencies, and thus, it provides a set of coefficients (in contrast to a single coefficient from correlation) which better describe the relationship between brain regions.

Pattern recognition methods based on machine learning algorithms have been successfully applied to fMRI datasets [20], [21], [22], [23], and one of the key features of this approach is that they are suitable for multivariate data. Unsupervised pattern recognition analysis consists of training a classifier with the data from a single group of subjects (usually the control group). This approach can be used to discriminate observations that do not belong to the same population from which the trained data was sampled. The one-class support vector machine (OC-SVM) was introduced by Schölkopf et al. [24] and Tax & Duin [25], and is founded on the structural risk minimization criteria [26]. This approach is suitable for medical research because it can be used to define statistical norms, and thus, to develop tools to support clinical diagnostic evaluation. In a recent study, Sato et al [27] illustrated how to build a normative database based on functional connectivity measures. Extending this approach, Mourão-Miranda et al. [28] applied OC-SVM to show that patient classification might be dealt as an outlier identification problem. In addition, OC-SVM can be used to obtain an index of how atypical an observation is, compared to a set of other observations. This feature might be useful in characterizing brain dysfunction, since this index can measure how abnormal a particular subject is when compared to a control group.

In this paper, considering that SCA provides a multivariate characterization (at different frequencies) of functional connectivity, we introduce a combination of SCA and OC-SVM to demonstrate that the functional connectivity between the posterior cingulate cortex (PCC) and dorsal anterior cingulate cortex (dACC) is not only different but abnormal in ADHD patients compared to subjects with typical development (TD). Moreover, we show that the functional connectivity patterns of patients with ADHD are more similar to those of young TD subjects, which reinforces the hypothesis that ADHD is related to abnormalities in brain maturation.

## Materials and Methods

### 2.1 Subjects

Data from 21 patients with ADHD (16 males, mean age 36.5 years, SD = 7.1, range 27–49 years), 21 age-matched TD subjects (10 males, mean age of 35.5 years, SD = 9.9, 20–50 years) and 21 young TD subjects (13 males, mean age of 16.7 years, SD = 4.1, 9–22 years) were used in this study. Patients were recruited from the New York University (NYU) School of Medicine Adult ADHD Program and controls were recruited through local media advertisements (as described in greater detail in Castellanos et al. [16]). To be included, patients had to meet ADHD lifetime criteria for Combined Type ADHD. To rule out Axis I psychiatric disorders, the Structured Clinical Interview for DSM-IV (SCID) and a semi-structured clinical interview were administered in patients and healthy controls, respectively. Both groups were also screened with the Symptom Checklist-90-Revised (SCL-90-R) [29] and were excluded if they reported: 1) lifetime or current history of psychotic, mood, or substance use disorders; 2) current history of anxiety disorders; 3) previous treatment with psychotropics except stimulants (for ADHD group only); or 4) history of any neurological or chronic organic illness. All subjects (or guardians) provided written informed consent prior to participating, as approved by the ethics committees of New York University (NYU) and the NYU School of Medicine. No statistical differences were found for age (p = 0.606, Mann-Whitney test) or sex (p = 0.112, Chi-square test with continuity correction) between the ADHD and TD age-matched groups. The average score of ADHD patients on the ADHD Self Report Scale [30] was 30.00 (s.d. = 9.14) and on the Adult ADHD Clinical Diagnosis Scale (ACDS) [31] was 7.58 (s.d. = 1.60). Stimulants were discontinued for at least 1 day before scanning for those patients who were taking medication.

The data used in this study are publically available via the 1000 Functional Connectomes Project database (http://www.nitrc.org/projects/fcon_1000/). Data from 20 ADHD patients and six TD age-matched subjects were included in the sample described by Castellanos et al. [16].

### 2.2 fMRI Acquisition

All subjects underwent a resting state scanning session, with instructions to relax with eyes open. One hundred ninety seven contiguous whole-brain EPI volumes were acquired in a Siemens 3.0 Tesla Allegra (TR = 2000 ms; TE = 25 ms; flip angle = 90, 39 slices, matrix = 64×64; FOV = 192 mm; voxel size = 3×3×3 mm, total scanning time of 6.58 minutes). A high resolution anatomical MRI was also acquired.

### 2.3 Image Preprocessing

The data was preprocessed using routines of FSL toolbox (www.fmrib.ox.ac.uk/fsl/). The images were processed for motion correction (using MCFLIRT routines, http://www.fmrib.ox.ac.uk/fsl/mcflirt/index.html), spatial normalization (MNI152 template, using FLIRT routines, http://fsl.fmrib.ox.ac.uk/fsl/flirt/, 12 degrees-of-freedom), spatial smoothing (Gaussian kernel, FWHM = 5 mm), temporal filtering (high-pass, 100 s cut-off, using SUSAN routines, http://www.fmrib.ox.ac.uk/fsl/susan/index.html). Further analyses were carried out using R platform (www.r-project.org). VAR modeling for coherence estimation was carried out using the “ar” routines of the “base” package and OC-SVM training was done using the “svm” routines in the “e1071” package.

### 2.4 Region-of-interest Selection

**Figure 1:**
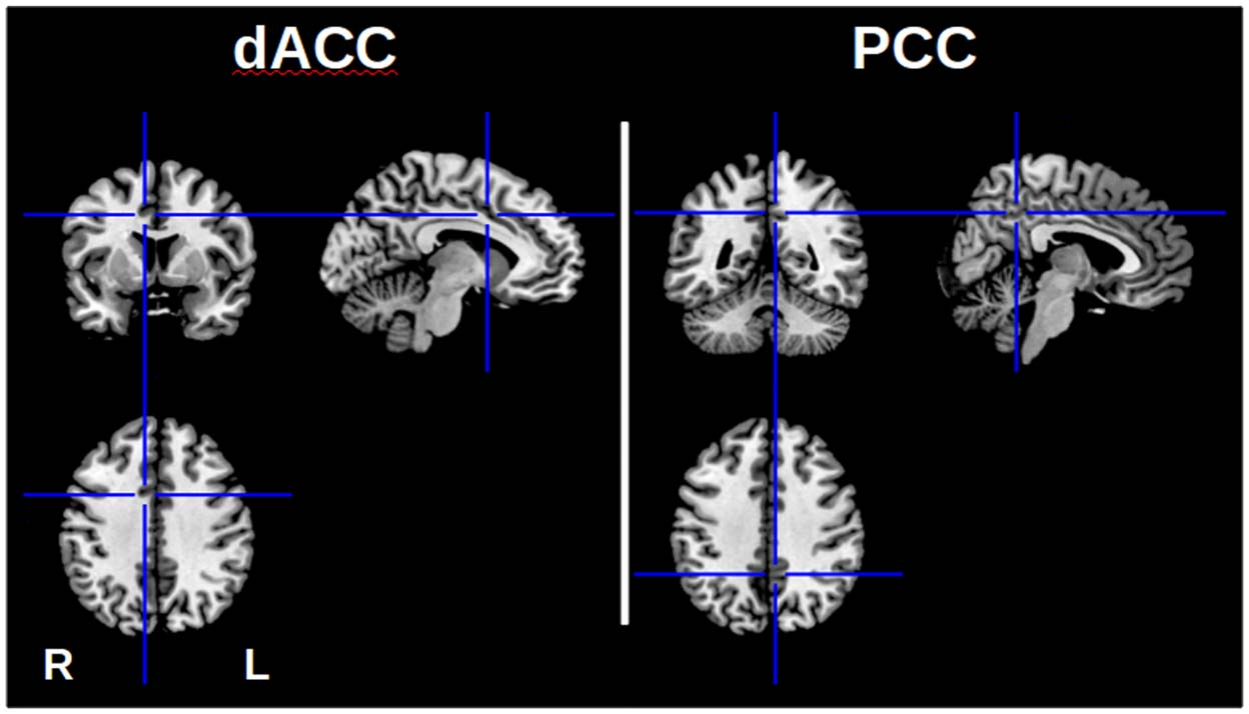
ROIs location for PCC and dACC. The coherence values between the BOLD signals from these two areas are used as predictors to the classifier.

Based on previous studies in literature, PCC and dACC were a priori defined as regions-of-interest (ROIs). The PCC ROI was defined using a spherical ROI of radius 7.5 mm centered at MNI coordinates (−5; −49; 40), based on Biswal et al. [32]. Similarly, dACC coordinates were (8; 7; 38), based on Weissman et al. [17]. Figure 1 depicts the ROI locations. All time series were normalized to mean zero and variance one.

### 2.5 Coherence Analysis

Functional connectivity between ROIs was quantified using spectral coherence analysis (SCA) [18], [19]. The reason for using SCA instead of conventional Pearson correlations are: i) SCA is more informative than correlation since it decomposes the correlation into different frequencies, ii) resting state networks have different spectral signatures, iii) correlation is univariate and coherence is multivariate (a vector of observations). In addition, SCA is a well established measure in signal processing literature and its properties and limitations are well known.

For each subject, SCA was calculated at each ROI (average time series of all voxels in a region as representative) over the whole resting state session, estimating the parameters of a vector autoregressive model (parametric SCA estimation), given by:
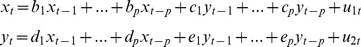
where 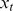 and 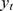 are the signals measured at time 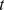 at the PCC and dACC, respectively, 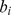
’s, 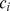
’s, 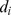
’s and 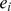
’s (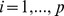) are coefficients and 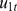 and 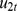 are white noise random errors. The lag order of the model was chosen by applying the Akaike Information Criterion [33], [34] independently for each subject. This selection is based on fitting VAR models of different orders (in this study, from 1 to 10) to the data, and then using the AIC formula. Based on this criterion, the best model is the one that minimizes AIC value. The lag order analysis was independently set for each individual due to the expected inter-subject variability and heterogeneity. However, the distribution of lag order did not differ significantly among patients with ADHD, age-matched TD and young TD subjects (Fisher exact test p = 0.147).

Consider the following matrix:
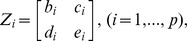
and



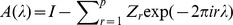
where *I* is the identity matrix and



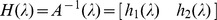



The coherence matrix is given by,
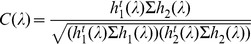
where 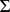 is the covariance matrix between 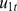 and 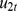. This is a symmetric matrix and the SCA measure is given by the off-diagonal values. The VAR model parameters were estimated using the Yule-Walker approach [19] and estimates for coherence were obtained. Coherence was sampled in 125 equally spaced frequencies 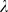 from zero to 0.25 Hz (which is the maximum frequency band range that can be analyzed in this study, considering the temporal sampling rate).

### 2.6 One-class Support Vector Machines

The foundations of the one-class support vector machine were developed by Schölkopf et al. [24], based on the statistical learning theory of Vapnik [26]. Support vector machine (SVM) classifiers are part of a set of methods for pattern recognition based on structural risk minimization, which theoretically yields high generalization power. The generalization power refers to the ability, once a training dataset is given, to extract some key features from this data in order to make predictions on new observations. One of the main advantages of SVM based approaches compared to other pattern recognition methods are their robustness in cases when each observation is composed of a large number of variables, even exceeding the sample size. Detailed information about SVM theory is described in Vapnik [26] and Schölkopf & Smola (2002) [35].

In the current study, we applied one-class SVM (OC-SVM), developed for the main purpose of defining normative classification rules in cases where the number of variables is large. By normative rules, we mean defining a target population and then predicting whether a new observation belongs to the population or not. The intuition behind OC-SVM is that “all positive examples are alike and each negative example is negative in its own way” (cf. first sentence of Leo Tolstoy's *Anna Karenina*). In the univariate case, the implementation of general normative rules is intuitive, since it can be based on building confidence intervals (not for the mean, but for an observation from the random variable of interest). The confidence interval can be obtained parametrically by assuming a probability distribution (e.g., Gaussian) or non-parametrically by using empirical quantiles. On the other hand, in cases where the number of variables is large (even greater than the sample size), the definition of these “multivariate quantiles” is not simple, and must be founded on mathematical and probability theory. Since the application of OC-SVM is not based on assuming two-classes a priori (e.g., controls and patients) but uses only the target group as the training data, it is considered an unsupervised classifier. Basically, the main idea of OC-SVM is to find a partition or subset based on the training data, such that the probability that a new observation (test data) is not contained by this subset is *p*. This parameter is defined a priori within the interval from 0 to 1. Note that *p* is the rejection rate for observations from the same population of the training data, and thus, from a clinical normative definition perspective, it is the expected false positive rate.

Actually, there are infinite ways to define multivariate quantiles, and thus, in order to establish the uniqueness of the solution, the rule is chosen by considering the solution providing the minimum probability volume. In the current study, we applied OC-SVM based on the radial basis function (RBF) kernel, which projects the data into a hypersphere, and OC-SVM works by classifying the data from the origin. In addition, one of the key features of OC-SVM is that it can provide a score, given by.
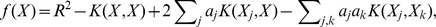
where 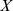 is the set of predictor features (in this case, the coherence values), the coefficients 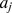 are obtained during the training of the classifier, 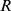 is the radius of the hypersphere and 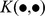 is the kernel function. The closer the observation is to the center of the hypersphere in feature space (the center is the most typical observation from the target) the higher is this score, and thus, the higher the probability that this observation belongs to the target population. Due to its complexity, the computational and mathematical foundations of OC-SVM formulations are not presented here. Further details about mathematical foundations and implementation can be found in the referenced literature.

The OC-SVM approach has been relatively well described in the machine learning literature and applications to neuroimaging databases can be found in Hardoon et al. [22], Song et al. [36], Sato et al. [37] and Mourão-Miranda et al. [28]. In this study, we applied OC-SVM to define an abnormality index for each subject. Since 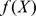 can be interpreted as a score function with higher values for typical observations, we define the abnormality index of a subject with features 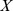 as 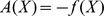. By definition, high values of 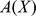 indicate abnormal observations, when compared to the target population.

Finally, it is important to mention that although two-class and multi-class SVM Vapnik [26] have been successfully applied in fMRI analyses, they are not suitable for identifying or quantifying abnormal multivariate patterns. These approaches are focused on classification and defining discriminative patterns and not on outlier or abnormality detection. This limitation was one of the main reasons that motivated the development of OC-SVM by Schölkopf et al. [24].

### 2.7 Processing Steps

The main idea in this study was to use OC-SVM to obtain an abnormality index of each subject to compare the dACC-PCC coherence measures between adult patients with ADHD, TD age-matched controls and young TD. Basically, the subjects of two groups (e.g., ADHD and TD age-matched controls) were assumed to be from the same single population. OC-SVM was trained using this mixed sample, the abnormality index was obtained for each subject from this sample and the distribution of this index was compared between the two original groups. These procedures guarantee that there is no systematic bias in the analysis, in favor of a specific group. Since OC-SVM was applied in a sample which is the mixture of two groups of the same size (21 subjects in each), the quantile parameter 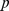 was set to 0.5 for all analyses. To avoid overfitting, the RBF kernel gamma parameter was not automatically tuned and was set to 1/125 (125 is the number of variables, i.e., the coherence at different frequencies), which is a standard heuristic [38].

The following steps were conducted:

- Step 1) Preprocessing of the EPI images of all subjects;
- Step 2) Extraction of the mean BOLD signal of each ROI, as representative time-series;
- Step 3) Calculation of coherence between the ROI signals;
- Step 4:
  - 4A) Train an OC-SVM classifier using the coherence of ADHD patients and TD age-matched controls as a single group. Obtain the abnormality index for all subjects;
  - 4B) Train an OC-SVM classifier using the coherence of TD age-matched controls and young TD subjects as a single group. Obtain the abnormality index for all subjects;
  - 4C) Train an OC-SVM classifier using the coherence of ADHD patients and young TD controls as a single group. Obtain the abnormality index for all subjects;
- Step 5) For each of the three classifiers in step 4, apply a Mann-Whitney test to assess the statistical significance of the difference of index values between groups. Since the hypothesis is that patients with ADHD will be abnormal (compared to TD subjects), which implies higher values of the abnormality index, the test is one-tailed (median of ADHD indices < median of TD indices). For comparison purposes, the evaluation between TD age-matched controls and young TD was also one-tailed.

### 2.8 Identification of Discriminative Frequencies

As described previously, the proposed abnormality index could be used to evaluate the coherence values of any given subject. Still, it does not provide any information as to why some subjects were identified as more abnormal than others. Thus, further analyses were carried out focusing on identifying frequencies which were more influential (regarding discrimination) in the analyses comparing ADHD patients vs TD age-matched controls and TD age-matched controls vs young TD subjects. All OC-SVM procedures of step 4 described in the previous section were repeated in a complementary analysis, in which the subjects’ abnormality indexes were recalculated, assuming a training set excluding each frequency at a time. We define the relevance coefficient of each frequency as.
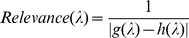
where 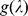 and 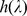 are the medians of the abnormality indices for the two groups being evaluated, when the respective coherence value at frequency 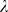 is not included in OC-SVM training. This procedure allowed the measurement of the effects of each frequency on the ability to discriminate the groups based on the influences of the abnormality indices. If this relevance coefficient increases when the coherence value at frequency 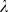 is excluded from analysis, this is evidence that this variable contains discriminative information to separate the groups.

### 2.9 Application of the Method to the ADHD-200 Sample

Although the main focus of the current study is the investigation of connectivity patterns and abnormality index in adults, we also performed the proposed analysis using the data of publicly released at the ADHD-200 Sample (http://fcon_1000.projects.nitrc.org/indi/adhd200/). The preprocessed data was released by The NeuroBureau (http://neurobureau.projects.nitrc.org/ADHD200/) and is available at the referred website. The preprocessing of fMRI data was performed using routines from the packages AFNI (afni.nimh.nih.gov/afni), FSL (www.fmrib.ox.ac.uk/fsl/) and processed at the Athena computational cluster at Virginia Tech’s ARC (www.arc.vt.edu). Data processing was based on: exclusion of the first four EPI volumes (in order to achieve MR steady-state), slice timing correction, deoblique dataset, reorientation of the volumes to RPI, correction for head motion (first volume as reference), masking the volumes to exclude voxels at non-brain regions, averaging the EPI volumes to obtain a mean functional image, co-registration of mean image to corresponding anatomic image of the subject, spatial normalization to standard space, removing effects of WM, CSF, motion and trend using linear multiple regression, temporal band-pass filter (0.009<f<0.08 Hz), spatial smoothing using a Gaussian kernel (FWHM = 6 mm). BOLD time series from dACC and PCC were extracted from ROIs at the same coordinates of the adult experiment as described in subsection 2.4. The ROIs were defined using the CC400 parcellation provided by the NeuroBureau.

Since the aim of this replication is a validation of the proposed approach, we calculated the abnormality index based on coherence and OC-SVM using exactly the same parameters described in subsection 2.4, 2.6 and 2.7. Similar to the adults analysis, the data was split in three subsamples: ADHD patients older than 15 years old (36 children; mean age = 17.25 years, s.d. = 1.58), age-matched typical developing children (87 children; mean age = 17.65 years, s.d. = 1.61), and young typical developing children (131 children; age between 12 and 15 years old; mean age = 13.47 years, s.d. = 0.87). The abnormality index of the subjects were calculated independently for each pairwise combination between the three groups, by following the approach described in subsection 2.7. All research carried out by ADHD-200 contributing sites was conducted under local IRB approval. The data was fully anonymized in compliance with HIPAA Privacy Rules.

## Results

**Figure 2:**
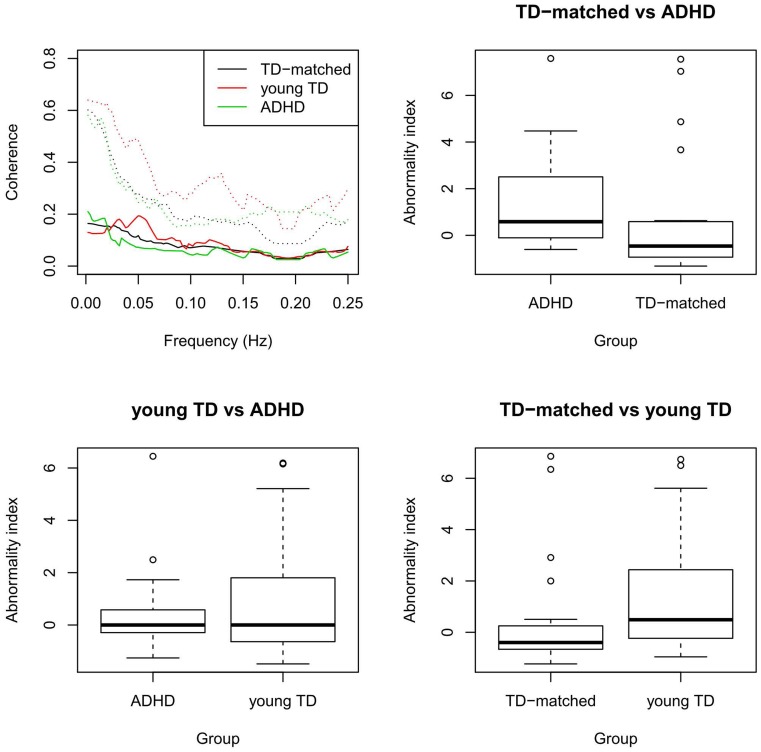
Typical development and ADHD patients’ median coherence (and 75% quantiles – dotted lines) and boxplots of the abnormality indexes of pairwise comparisons between groups. These indexes show that ADHD patients have a similar pattern of young TD and both differ from TD-matched controls.

Figure 2 depicts the median coherence (and 75% quantile) of each group. The abnormality indices from subjects for each combination of the two groups are also presented in Figure 2. Note that there is strong evidence that the median index of patients with ADHD is greater than in TD age-matched subjects (p = 0.014), there is no difference between ADHD and young TD indexes (p = 0.480), and the median index of young TD is greater than TD age-matched subjects (p = 0.016). Although some studies suggest that sex may not affect resting state fMRI metrics [39], we also assessed the potential confounding effect of sex on our findings. Mann-Whitney tests were performed to assess sex effects and quadratic regression (with age as a predictor and abnormality index as a response) to test age effects. No significant effects of sex (p = 0.143) or age (p = 0.589) were found.

**Figure 3:**
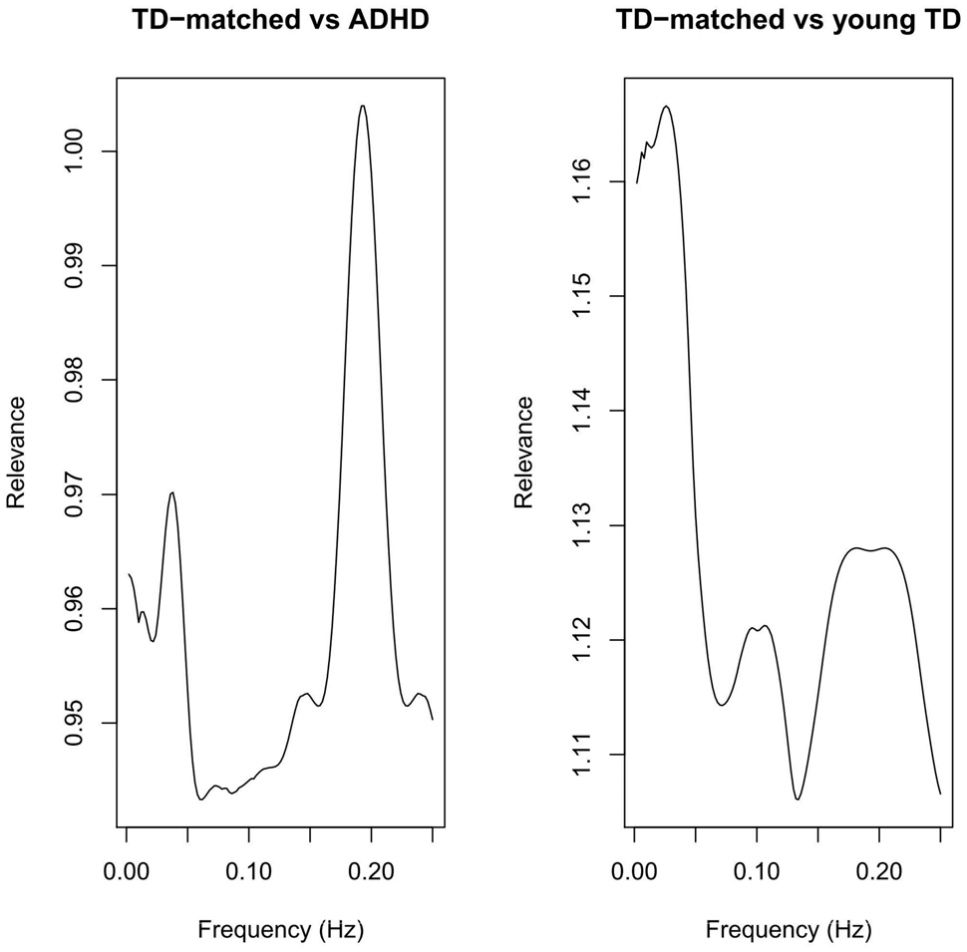
Discriminative relevance of each frequency in groups comparison. The frequencies below 0.05 and around 0.20 Hz contain most discriminative information.

The relevance of each frequency in group discrimination is shown in Figure 3. This figure suggests that although OC-SVM uses the information of all frequencies in an unsupervised fashion, low frequencies below 0.05 Hz and around 0.20 Hz are the most relevant for discriminating between ADHD patients from TD age-matched controls and between the older and younger TD. This result supports reinforces the conclusion that patients with ADHD and young TD have a more similar pattern, since both groups share the same features that differentiate them from the TD age-matched controls.

**Figure 4:**
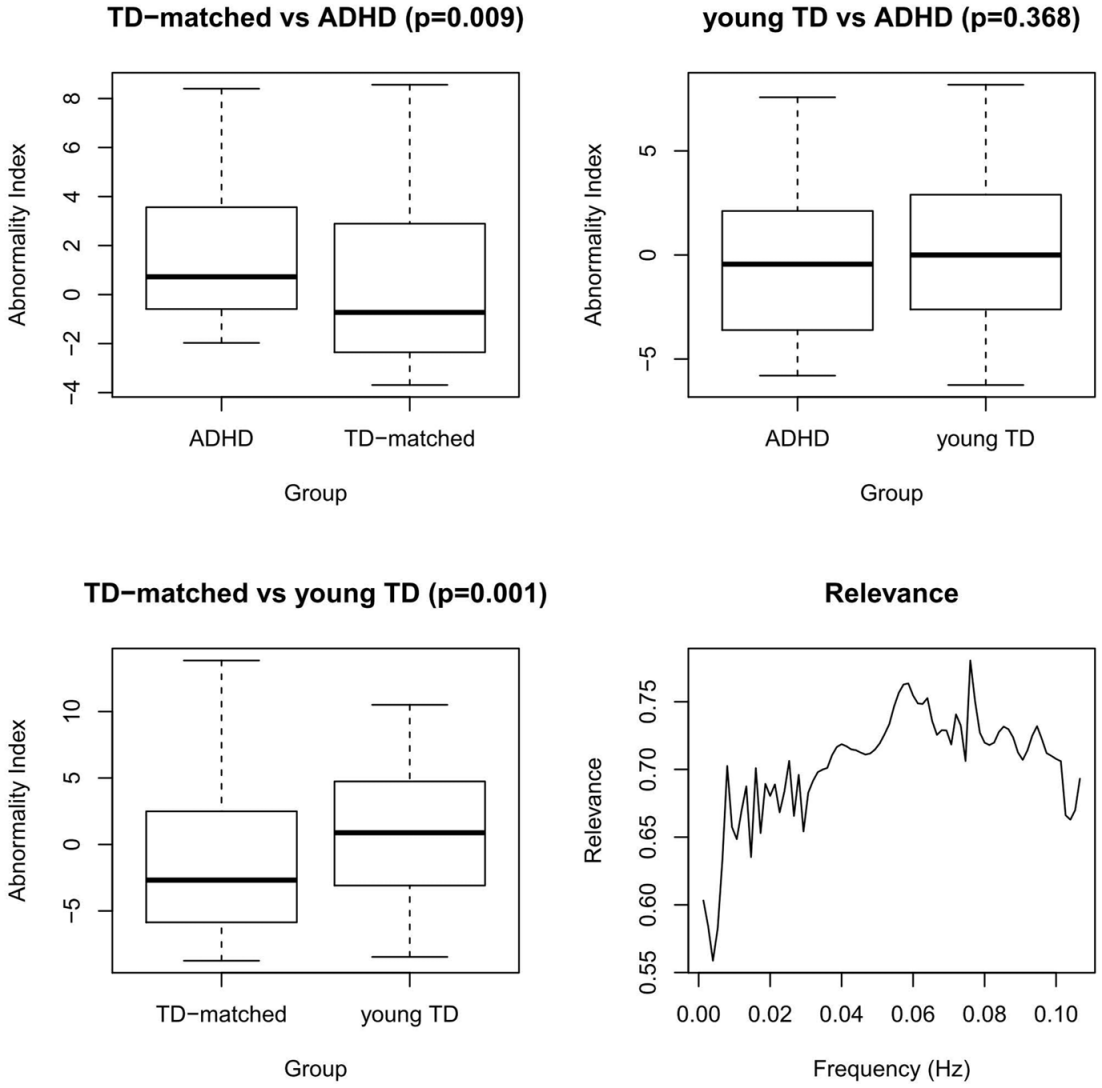
ADHD-200 Sample: Boxplots of the abnormality indexes of pairwise comparisons between groups at different ages. Bottom-right: relevance of each frequency in discriminanting ADHD patients from TD age-matched controls. These findings are very similar to the ones obtained in the adults dataset.

Figure 4 shows the results from the application of the proposed approach to the ADHD-200 sample. Note that the results is analogous to the ones obtained in the adult sample: ADHD patients present abnormal connectivity pattern when compared to age-matched TD (p = 0.009), age-matched TD and young TD patterns are different (p = 0.001), and ADHD and young TD have a more similar patern (p = 0.368). Furthermore, low frequencies around 0.05 Hz are the most relevant for discriminating between ADHD patients from TD age-matched controls.

## Discussion

This is the first study using OC-SVM and spectral coherence measures to demonstrate that resting state functional connectivity between PCC and dACC is abnormal (and not only different) in patients with ADHD when compared to TD age-matched controls and that these abnormal coherence patterns of ADHD patients are more similar to the patterns observed in young subjects with typical development. To address these questions, we introduced a multivariate approach to define an abnormality index for any subject.

We note that this was not an exploratory study using massive-multivariate classifiers with the objective of obtaining maximum accuracy in discriminating groups. The aims of this study were threefold: i) to demonstrate that ADHD patients have abnormal dACC-PCC coherence patterns (in a multivariate sense); ii) to identify the abnormal frequencies; and iii) to obtain evidence addressing the brain maturation hypothesis of ADHD.

Our finding of abnormal PCC/dACC coherence between ADHD patients and TD age-matched controls provides supports the use of this approach as a potential tool for investigating ADHD. Until recently, most neurobiological studies of ADHD investigated brain regions encompassing the prefrontal–striatal and mesolimbic circuits involved in executive functions and inhibitory control [6], [7]. However, beyond inhibitory control dysregulation, patients with ADHD also present substantial impairments in attentional performance [40], which suggests alternative brain circuits may also be involved in the pathophysiology of ADHD. There are indications showing that deficits in deactivation of the default-mode network underlie lapses in attention [17]. In this regard, ADHD has been reported to be associated with dysfunction in fronto-default-mode networks [16]. Specifically, the functional connectivity between dACC and posterior components of the default-mode network was decreased in 20 adults with ADHD compared to 20 healthy volunteers [16].

Interestingly, we did not observe any statistically significant difference in the coherence index of ADHD patients compared to young TD subjects. Whether ADHD is a disorder related to a delay in brain maturation or whether it represents a frank derangement of typical development has remained an unsettled question since its earliest descriptions [41]. Several neuroimaging studies have found similarities in functional brain patterns between patients with ADHD and younger TD controls [42], [43]. Interestingly, the NIMH longitudinal study support both types of conclusions. In comparing 223 children with ADHD and 223 typically developing controls, Shaw et al. [44] found that cortical thickness maturation trajectories were similar in both groups. However, children with ADHD reached peak thickness later than typically developing controls in most areas of the brain [44]. However, in an earlier analysis with nearly the same data, Shaw et al. [45] reported that overall cortical thickness was significantly reduced in the group with ADHD, on average, compared to healthy controls. Our results are qualitatively consistent with the concept that ADHD is characterized by a delay in brain maturation rather than a frank derangement of typical development. In addition, Figure 2 (top, right) suggests that the variability of the index in the ADHD group is larger than in TD age-matched controls, showing that the patient group is more heterogeneous. This finding is also in agreement with other studies in the literature, which show that the variability of neuropsychological and neurophysiological measures is larger in patients with ADHD [46], [47]. In addition, similar findings are found when applying the proposed approach to the ADHD-200 children sample. All these results are complementary and emphasize that ADHD is related to deviations during neurodevelopment.

A limitation of the proposed approach is that it was developed to deal only with two regions of interest, in this case, dACC and PCC. Technically, the method could be extended to the case of three or more brain regions by considering the coherence between all pairwise combinations of ROIs as features in OC-SVM. However, in practice, this would lead to a massive increase in the number of variables. Since the samples for most fMRI studies are relatively small (less than 100), the inclusion of too many variables would decrease the performance of OC-SVM, due to the “curse” of dimensionality and the presence of irrelevant variables. In addition, the successful application of OC-SVM is strongly dependent on the signal-to-noise ratio of the observations. This was the main reason for using the ROI average signal as regional representative, before calculating coherence values. Actually, this is a limitation of using an unsupervised classifier, since all variables are assumed to have the same relevance in the calculation of the abnormality index. However, it is the unsupervised nature of the classifier which ensures that the analysis of the abnormality index would not be biased by the selection of the target population (ex: if only the control group was chosen as the target), since both ADHD and TD groups are mixed in a single group, before extracting the indexes of each subject.

The results of this study suggest that low frequencies below 0.05 and around 0.20 Hz are most relevant to ADHD. This frequency characterization was relevant to show that the features that led ADHD patients to be classified as abnormal compared to TD age-matched controls, were the same features that were abnormal in young TD. However, the neurophysiological meaning of this finding is still an unanswered question. Although there are some studies investigating changes in the amplitude of low frequency fluctuations [48], there are few studies with systematic investigation into the frequency domain features of functional connectivity in ADHD [49].

Future studies should explore and describe the frequency properties of functional connectivity in ADHD and also evaluate the proposed approach in other samples of ADHD patients. The inclusion of patients with different psychiatry disorders would also be important to investigate whether these findings would be specific for ADHD.
